# Correct names for some of the closest relatives of *Carica papaya*: A review of the Mexican/Guatemalan genera *Jarilla* and *Horovitzia*

**DOI:** 10.3897/phytokeys.29.6103

**Published:** 2013-11-19

**Authors:** Fernanda Antunes Carvalho, Susanne S. Renner

**Affiliations:** 1 Systematic Botany and Mycology, Ludwig-Maximilians-Universität München, Menzinger Strasse 67, D-80638 Munich, Germany

**Keywords:** Caricaceae, nomenclature, epitypification, papaya sister clade

## Abstract

Using molecular data, we recently showed that *Carica papaya* L. is sister to a Mexican/Guatemalan clade of two genera, *Jarilla* Rusby with three species and *Horovitzia* V.M. Badillo with one. These species are herbs or thin-stemmed trees and may be of interest for future genomics-enabled papaya breeding. Here we clarify the correct names of *Jarilla heterophylla* (Cerv. ex La Llave) Rusby and *Jarilla caudata* (Brandegee) Standl., which were confused in a recent systematic treatment of *Jarilla* (McVaugh 2001). We designate epitypes for both, provide weblinks to type specimens, a key to the species of *Jarilla* and *Horovitzia*, and notes on their habitats and distribution.

## Introduction

The family Caricaceae Dumort. comprises 34 species and one formally named hybrid in currently six genera. A molecular phylogeny that included all species revealed that *Carica papaya* L. (the only species in the genus *Carica*) is sister to a clade of four species endemic to Mexico and Guatemala (Carvalho and Renner 2012). The discovery that the closest relatives of *Carica papaya* are three herbs in the genus *Jarilla* Rusby and a thin stemmed tree, *Horovitzia cnidoscoloides* (Lorence & R. Torres) V. M. Badillo, has implications for plant breeders, who have so far tried in vain to cross papaya with tree species in the genus *Vasconcellea* A. St.-Hil., known as the highlands papayas. To facilitate communication among researchers from different fields, and since full-genome sequencing of the species of *Jarilla* and *Horovitzia* is ongoing (R. Ming, Urbana-Champaign, personal communication, Aug. 2013), we here provide a conspectus of the four species that are the closest relatives of papaya and clean up a nomenclatural confusion involving two names in the genus *Jarilla*.

We start with the nomenclatural issues, then provide a key to the four species, and end with brief comments on the range and habitat of each species.

## Nomenclature of *Jarilla*

**Figure 1.**
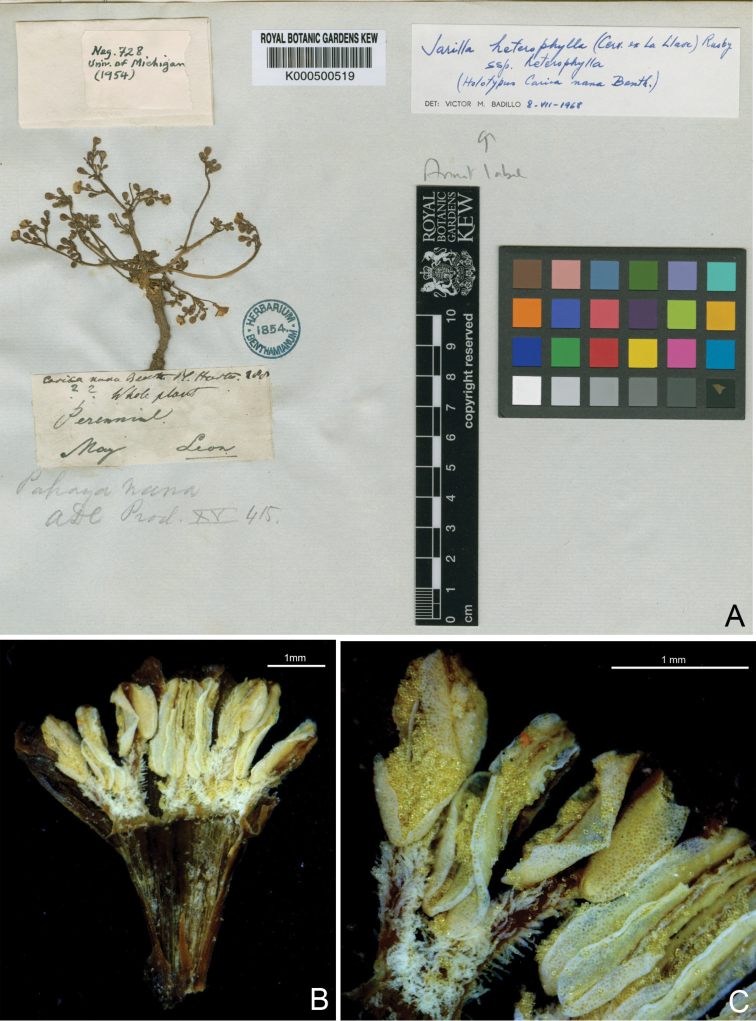
Holotype of *Carica nana* Benth. **A** Specimen in K (http://www.kew.org/herbcatimg/202388.jpg ) **B** Photo of an opened flower showing the arrangement of the anthers and the pistillode (arrow) **C** Close-up of the anthers. Filaments are densely covered by moniliform trichomes. **B** and **C** were taken by the first author in K.

Pablo de La Llave (1832), a director of the National Museum of Natural History of Mexico, was the first to describe one of the unusual herbaceous Caricaceae that are today placed in *Jarilla*. He had access to fruiting specimens only and based his description of the flowers on notes made by Vicent Cervantes, a professor of botany in Mexico City and one of the founders of that city’s botanical garden in 1788. La Lave gave his new species the epithet *“heterophilla”* [sic] to refer to its variably shaped leaves. To mark the distinctness of the new species, he placed it in a separate genus, *Mocinna*, honoring the Mexican naturalist José Mariano Mociño. Unfortunately, this overlooked that Lagasca in 1816 had already described an Asteraceae genus by that name. Soon thereafter, George Bentham (1839) described the same species as *Carica nana*, based on an unnumbered Hartweg specimen (Fig. 1) collected in 1836 in Léon (Guanajuato, Mexico). The holotype at K (Fig. 1) bears the number *288* on its label, a number corresponding to the page of *Plantae Hartwegianae* on which *Carica nana* was described. Diaz-Luna and Lomeli-Sención (1992), in their revision of *Jarilla*, cite this collection as *Hartweg 255* (K), probably due to a misreading of 288 for 255.

**Figure 2.**
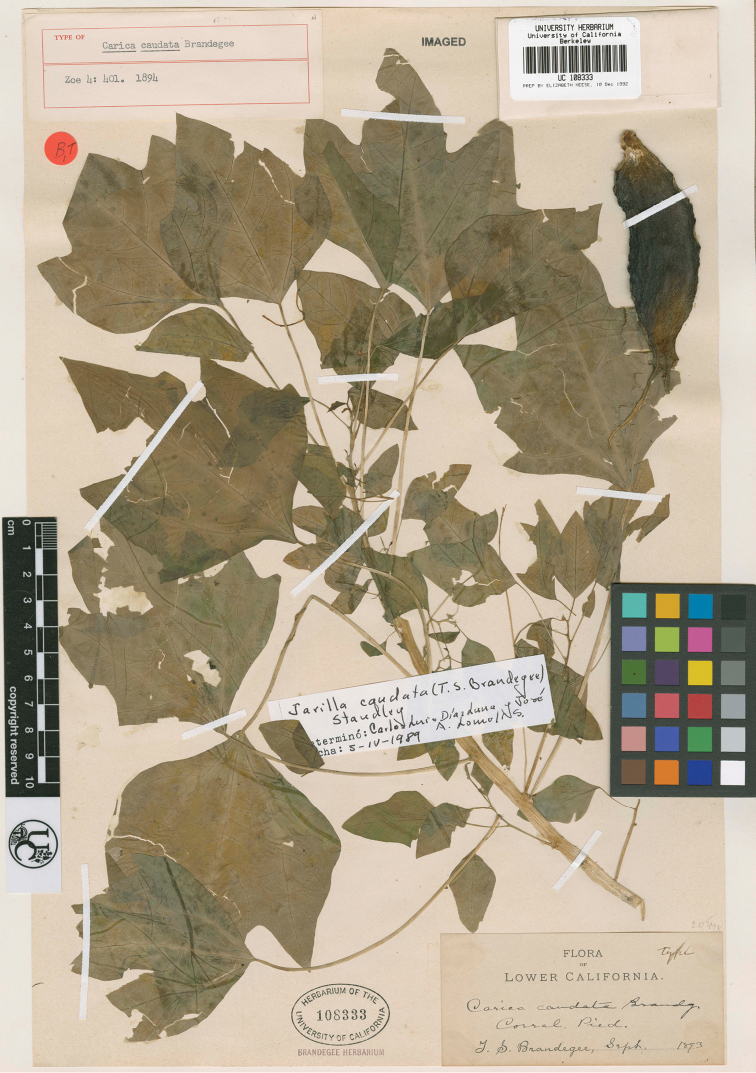
Holotype of *Carica caudata* Brandegee (http://ucjeps.berkeley.edu/new_images/UC108333.jpg )

**Figure 3.**
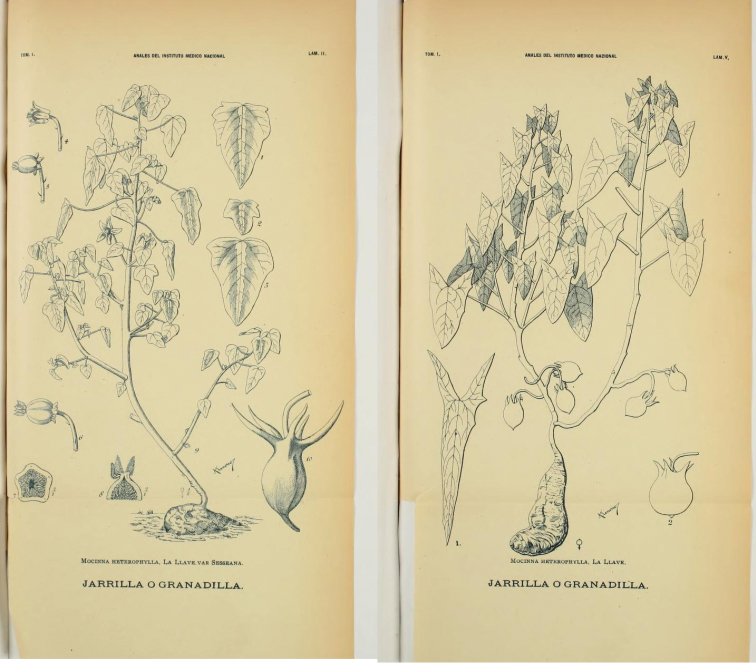
The two varieties of *Mocinna heterophylla* La Llave. **Left plate** shows the lectotype of *Mocinna heterophylla* var. *sesseana* Ramírez. **Right** **plate** shows the neotype of *Mocinna heterophylla* var. *heterophylla*, both designated by Díaz and Lomeli-Sención (1992). Plates reproduced from Ramírez (1894).

The second herbaceous Caricaceae species was named in March 1894 by Townshend S. Brandegee, who described *Carica caudata* from the Cape region of Baja California, Mexico, based on a plant he collected the year before (Fig. 2). In August of the same year, José Ramírez, unaware of Brandegee’s publication, described a new variety of the first herbaceous Caricaceae, *Mocinna heterophylla* La Llave, naming it varietas *sesseana*, based on living plants from Guanajuato and Jalisco. Unfortunately, he appears to have made no herbarium specimens, but only two beautiful plates showing the typical variety and var. *sesseana* (Fig. 3). Comparison of the plate of var. *sesseana* and the holotype of *Carica caudata* leaves no doubt that these names refer to the same species, and we therefore agree with previous assessments (Diaz-Luna and Lomeli-Sención 1992, Badillo 1993) that they are synonyms.

Realizing that *Mocinna* La Llave was a younger homonym of *Mocinna* Lag., Henry Hurd Rusby (1921) proposed the substitute name *Jarilla*, derived from the Spanish vernacular name Jarrila, for *Mocinna heterophylla*. He also up-ranked var. *sesseana* as a separate species, *Jarilla sesseana* (Ramírez) Rusby. We agree with Diaz-Luna and Lomeli-Sención (1992) and McVaugh (2001) that Rusby’s publication of the substitute name *Jarilla* meets the requirement for valid publication and that Ivan M. Johnston’s (1924) slightly later publication of the name *Jarrilla* (the correct Spanish spelling) to replace *Mocinna* is a superfluous name. At around the same time, Standley (1924) realized that *Carica caudata* Brandegee belonged in *Jarilla* and was in fact an older name for *Jarilla heterophylla* var. *sesseana* Ramírez (= *Jarilla sesseana* (Ramírez) Rusby), and he accordingly changed the name to *Jarilla caudata*. He also described a third herbaceous species of Caricaceae, *Jarilla chocola* Standley, based on two collections made in 1935 from Sonora, Mexico (Standley 1937).

Thus, by 1937 it was clear there were three species of *Jarilla* and also what their correct names were. In their revision of the genus, Diaz-Luna and Lomeli-Sención (1992) designated plate II of Ramírez (1894; our Fig. 3 left-hand plate) as the lectotype of *Jarilla heterophylla* var. *sesseana* and plate V as the neotype of var. *heterophylla* (our Fig. 3 right-hand plate). Unfortunately, the most recent study of *Jarilla*, that of Rogers McVaugh (2001), synonymized the two taxa distinguished by Ramírez. This error is surprising given the different leaves and fruits of Ramírez’s two varieties (our Fig. 3), and indeed McVaugh seems to have been aware he might be making a mistake because he writes (2001: 469), “In the following I have drawn heavily upon the work of Diaz-Luna and Lomelí-Sención, whose personal observations of these interesting species greatly increased our knowledge of them, and have indeed provided almost all the available information about the living plants. Errors introduced here, as a result of faulty translation or interpretation of the work of these authors, or otherwise, are solely my responsibility.”

**Figure 4.**
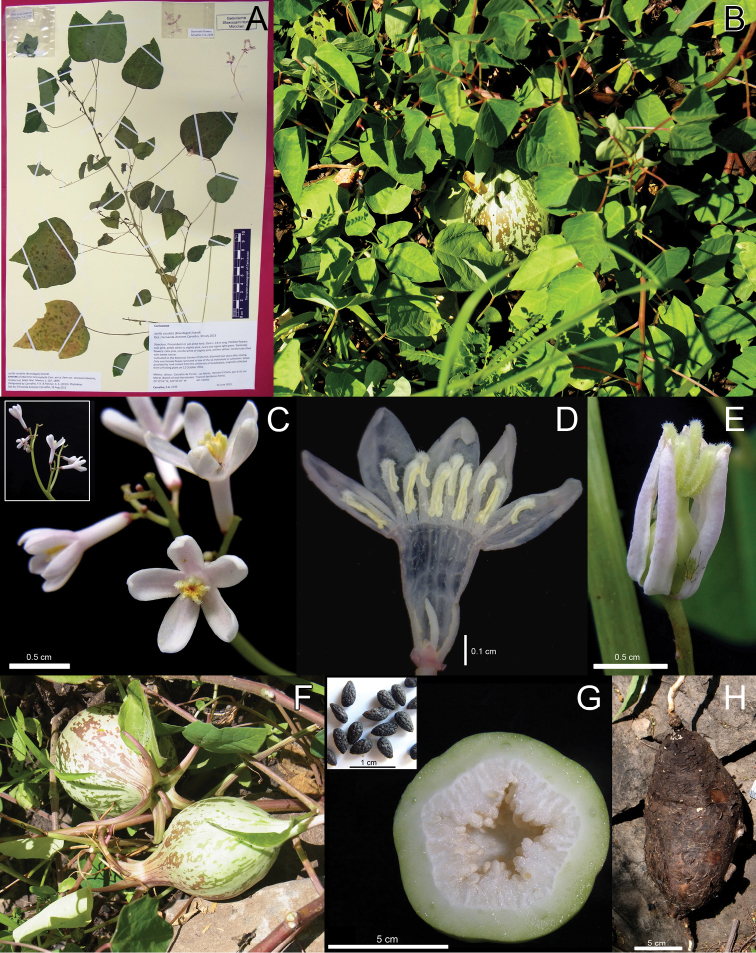
*Jarilla caudata* (Brandegee) Standl. **A** Epitype of *Mocinna heterophylla* La Llave (*F.A. Carvalho 2240*, M) **B** Habit **C** Male inflorescence **D** Staminate flower. **E** Pistillate flower **F** Fruits **G** Ovary unilocular and seeds **H** Tuber.

**Figure 5.**
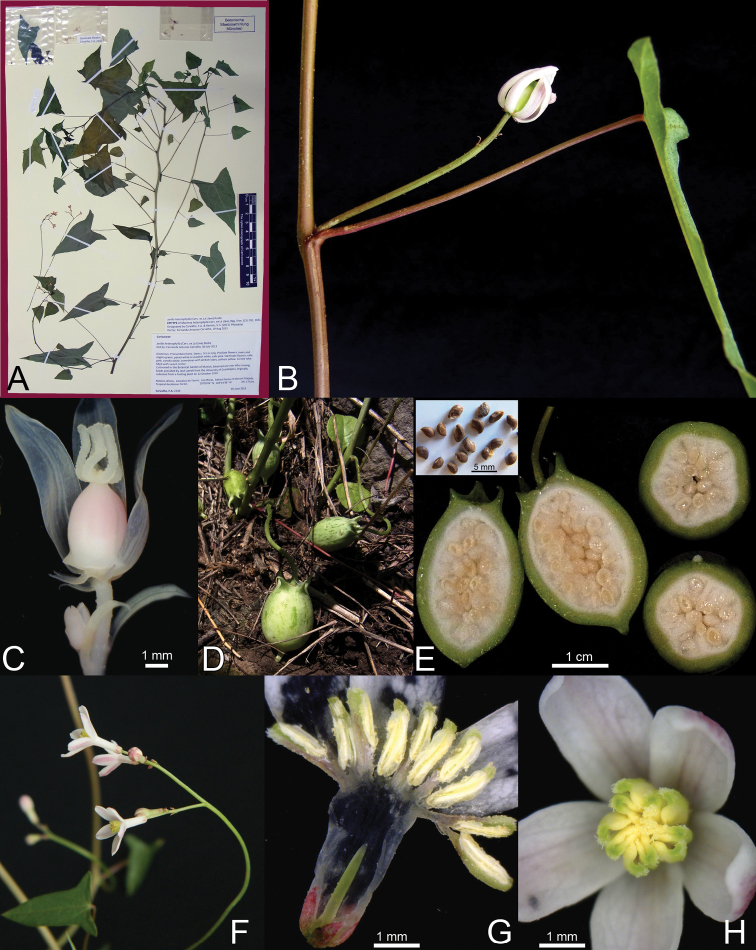
*Jarilla heterophylla* (Cerv. ex La Llave) Rusby. **A** Epitype of *Mocinna heterophylla* La Llave var. *sesseana* Ramírez (*F.A. Carvalho 2239*, M) **B** Female inflorescence (uniflora). **C** Female flower showing the short appendages at the base of the ovary **D–E** Fruits and seeds **F** male inflorescence **G–H** Staminate flowers.

We agree with Diaz-Luna and Lomeli-Sención (1992) and the earlier workers cited above that *Jarilla heterophylla* var. *heterophylla* is the oldest name for Bentham’s *Carica nana*, while var. *sesseana* is a younger synonym of *Carica caudata*. We have accordingly up-dated the names of our previous *Jarilla heterophylla* and *Jarilla nana* sequences in GenBank (Carvalho and Renner 2012; all of which are vouchered). Together, the descriptions of Ramírez (1894), Brandegee (1894), Rusby (1921), Johnson (1924), Standley (1924), and Diaz-Luna and Lomeli-Sención (1992) provide a clear idea of the morphological distinctions of the two species: *Jarilla caudata* has rounded to ovate or deltoid (never hastate) leaves, c. 1 cm (rarely longer) male flowers, and 10 cm long fruits that are narrowed at the base with five horn-like appendages, each 3–6 cm long (Fig. 4). *Jarilla heterophylla* has hastate leaves, 0.5 cm long male flowers, and c. 3 cm long fruits with short and thick appendages as shown in Fig. 5.

To fix the usage of the two names more reliably, we below designate epitypes to serve as interpretative specimens for plates II and V of Ramírez (1894), following Art. 9.8 of the Melbourne Code (McNeill et al. 2012). The plates published by Ramírez fail to include staminate and pistillate flowers for both species and therefore do not precisely fix the application of the names of these dioecious species. In addition, physical specimens also can help in evolutionary studies because they can yield DNA that may be used in future comparisons. We chose as epitypes complete male and female specimens from the same population. The epitypes are deposited in M. Isoepitypes of *Mocinna heterophylla* Cerv. ex La Llave var. *sesseana* (=*Jarilla caudata* (Brandegee) Standl.) are in MEXU and NY. Isoepitypes of *Mocinna heterophylla* Cerv. ex La Llave (= *Jarilla heterophylla* (Cerv. ex La Llave) Rusby) are in MEXU, NY and K.

The four species in the *Jarilla*/*Horovitzia* clade can be distinguished from all other Caricaceae and from each other, using a combination of the plastid markers *trnL-trnF* and *psbA-trnH* (Carvalho and Renner 2012; GenBank accessions JX091966, JX091977, JX091975, JX091978, JX092054, JX092064, JX092065, JX092066).

### Key to the species of *Jarilla* and *Horovitzia*

**Table:** 

1a	Small tree, completely covered by stinging hairs	*Horovitzia cnidoscoloides*
1b	Herb, glabrous or pubescent, but never with stinging hairs	2
2a	Erect herb. Leaves lobate, rarely entire. Ovary and mature fruits with 5 longitudinal wings. Female flowers 7–9 mm long. Male flowers 5–9 mm long	*Jarilla chocola*
2b	Procumbent herb, sometimes using understory plants for support. Leaves entire, rarely lobed. Ovary and young fruits with 5 basal appendages, but not winged. Female flowers 5–15 mm long. Male flowers 4-12 mm long	3
3a	Mature fruits 6–30 cm long with 5 horn-like basal appendages 3–6 cm long. Seeds black, 4–5.5 mm long. Male flowers in general >1 cm (1–1.7 cm)	*Jarilla caudata*
3b	Mature fruits 2–4 cm long with 5 curved basal appendages 0.5–2 cm long. Seeds light brown, 2.5–3.5 mm long. Male flowers in general <1 cm (0.3–0.8 cm)	*Jarila heterophylla*

### Epitypification and comments on morphology and habitats

#### Horovitzia cnidoscoloides

(Lorence & R. Torres) V.M. Badillo, Rev. Fac. Agron. (Maracay) 43: 104. 1993.

http://species-id.net/wiki/Horovitzia_cnidoscoloides

Carica cnidoscoloides Lorence & R. Torres, Syst. Bot. 13(1): 107–109, f.1. 1988.

##### Type:

Mexico. Oaxaca: Ixtlan, Sierra de Juárez, 9 March 1986, *R. Torres & P. Teonorio 8168* (holotype: MEXU, a photo in GUADA; isotypes: BM, MO [MO-193213], NY[00112155]). Mexico. Oaxaca. Type locality, 25 May 1883, *T. Cedillo & Lorence 2347* (paratype: MEXU, a photo in GUADA, a duplicate in MO); 4 Ago 1985, *Lorence et al. 4733* (paratype: MEXU, a duplicate in BM); 9 Mar 1985, *C. Torres & L. Tenorio 8167* (paratype: MEXU); 27 Ago 1986, *C. Torres & L. Tenorio 8760* (paratype: MEXU).

*Horovitzia cnidoscoloides* is a small tree, 0.5–4 m tall endemic to Sierra de Juarez in Oaxaca, Mexico. It occurs in cloud forests from 800 to 1600 m above sea level. Unusual features are subcapitate stigma, and stinging hairs covering the entire plant.

#### Jarilla chocola

Standl. Publ. Field Mus. Nat. Hist., Bot. Ser. 17: 200. 1937.

http://species-id.net/wiki/Jarilla_chocola

##### Type.

Mexico. Sonora: Chihuahua, Guasarema, Rio Mayo, 10 August 1936, *H. S. Gentry 2366* (holotype: F; isotypes: GUADA photo, K [K000500520], S [S-G-3434]). Mexico. Sonora: San Bernardo, Rio Mayo, 14 August 1935, *H. S. Gentry 1624* (paratype: F, duplicates in MEXU and K [000500521], a photo in GUADA).

*Jarilla chocola* is an erect herb, with mostly lobate leaves and fruits with 5 longitudinal wings. The species occurs at low altitudes (100–1300 m) along the Pacific Coast from Sonora to El Salvador.

#### Jarilla caudata

(Brandegee) Standl., Contr. U.S. Natl. Herb. 23(4): 853. 1924.

http://species-id.net/wiki/Jarilla_caudata

Fig. 4

Carica caudata Brandegee, Zoe 4: 401. 1894. Type: Mexico. Baja California Sur: Corral de Piedra, September 1893, *Brandegee s.n.* (holotype: UC[UC108333]).
Mocinna heterophylla var. *sesseana* Ramírez, Anales Inst. Med.-Nac. Mexico 1: 207. 1894. Type: Plate II of Ramírez, 1894 (lectotype designated by Diaz-Luna and Lomeli-Sención 1992: 81). Mexico, Jalisco, Zacoalco de Torres, Las Moras, 5 June 2013, *F. A. Carvalho 2239* (epitype, designated here: M; isoepitypes: MEXU, NY).
Jarilla sesseana (Ramírez) Rusby, Torreya 21: 47. 1921.

##### Remarks. 

*Jarilla caudata* is morphologically and phylogenetically closely related to *Jarilla heterophylla*. Their main distinguishing features are the fruits, which in *Jarilla caudata* can attain a length of 30 cm, having a smooth surface and 5 long, horn-like appendages (3–6 cm long). Other differences are given in the key. The species occurs in deciduous forests and fields of Baja California and central Mexico from 1500 to 1800 m above sea level.

#### Jarilla heterophylla

(Cerv. ex La Llave) Rusby, Torreya 21(3): 50. 1921.

http://species-id.net/wiki/Jarilla_heterophylla

Fig. 5

Mocinna heterophylla Cerv. ex La Llave, Reg. Trim. 1(3): 351. 1832. Type: Plate V of Ramírez, 1894 (neotype, designated by Diaz-Luna and Lomeli-Sención 1992: 88). Mexico, Jalisco, Zacoalco de Torres, Las Moras, 5 June 2013, *F. A. Carvalho 2240* (epitype, designated here: M; isoepitypes: MEXU, NY, K).
Carica nana Benth., Pl. Hartw. 288. 1849. Type: Mexico. Guanajuato, Leon, *K. T. Hartweg s.n*. (holotype K [K000500519]; isotype: G-DC n.v.).
Papaya nana (Benth.) A. DC., Prodr. 15(1): 415. 1864.
Jarilla nana (Benth.) McVaugh, Fl. Novo-Galiciana 3: 475. 2001.

##### Remarks.

For differences from *Jarilla caudata* see under that species and in the key. *Jarilla heterophylla* occurs in oak forests, deciduous forests, and abandoned fields of central Mexico at 1500 to 2700 m above sea level.

### Weblinks to type specimens

*Carica caudata* Brandegee, holotype:

http://ucjeps.berkeley.edu/new_images/UC108333.jpg [accessed 30.07.2013]

*Carica cnidoscoloides* Lorence & R. Torres, isotypes:

http://www.tropicos.org/Image/11116 [accessed 11.08.2013]

http://sweetgum.nybg.org/vh/specimen.php?irn=707429 [accessed 11.08.2013]

*Carica nana* Benth., holotype: http://www.kew.org/herbcatimg/202388.jpg [accessed 30.07.2013]

*Jarilla chocola* Standl., isotypes:

http://apps.kew.org/herbcat/getImage.do?imageBarcode=K000500520 [accessed 11.08.2013]

http://andor.nrm.se/kryptos/fbo/kryptobase/large/S-G-003001/S-G-3434.jpg [accessed 11.08.2013]

*Mocinna heterophylla* Cerv. ex La Llave, epitype:

http://herbaria.plants.ox.ac.uk/bol/caricaceae [accessed 11.10.2013]

*Mocinna heterophylla* var. *sesseana* Ramírez, epitype:

http://herbaria.plants.ox.ac.uk/bol/caricaceae [accessed 11.10.2013]

## Supplementary Material

XML Treatment for Horovitzia cnidoscoloides

XML Treatment for Jarilla chocola

XML Treatment for Jarilla caudata

XML Treatment for Jarilla heterophylla
